# Refined Mapping of a Quantitative Trait Locus on Chromosome 1 Responsible for Mouse Embryonic Death

**DOI:** 10.1371/journal.pone.0043356

**Published:** 2012-08-16

**Authors:** Magalie Vatin, Gaetan Burgio, Gilles Renault, Paul Laissue, Virginie Firlej, Françoise Mondon, Xavier Montagutelli, Daniel Vaiman, Catherine Serres, Ahmed Ziyyat

**Affiliations:** 1 Université Paris Descartes, Institut Cochin Inserm U1016 CNRS UMR 8104, Paris, France; 2 Institut Pasteur, Unité de Génétique des Mammifères, Paris, France; 3 Department of Genetics, Menzies Research Institute, University of Tasmania, Hobart, Australia; 4 Unidad de Genética, Facultad de Medicina, Universidad Del Rosario, Bogota, Colombia; Nagoya University, Japan

## Abstract

Recurrent spontaneous abortion (RSA) is defined as the loss of three or more consecutive pregnancies during the first trimester of embryonic intrauterine development. This kind of human infertility is frequent among the general population since it affects 1 to 5% of women. In half of the cases the etiology remains unelucidated. In the present study, we used interspecific recombinant congenic mouse strains (IRCS) in the aim to identify genes responsible for embryonic lethality. Applying a cartographic approach using a genotype/phenotype association, we identified a minimal QTL region, of about 6 Mb on chromosome 1, responsible for a high rate of embryonic death (∼30%). Genetic analysis suggests that the observed phenotype is linked to uterine dysfunction. Transcriptomic analysis of the uterine tissue revealed a preferential deregulation of genes of this region compared to the rest of the genome. Some genes from the QTL region are associated with VEGF signaling, mTOR signaling and ubiquitine/proteasome-protein degradation pathways. This work may contribute to elucidate the molecular basis of a multifactorial and complex human disorder as RSA.

## Introduction

Embryonic development in mammals begins from the female and male interaction which leads to the oocyte fertilization. After 5 to 6 cell divisions inside the zona pellucida, the blastocyst undergoes its development conducing to the implantation in the uterine tissue. The external cells of the blastocyst develop into the placenta, a pivotal organ which allows immune tolerance, bidirectional foeto-maternal exchanges and crucial synthesis of gestational hormones [1]. All these biological processes are required for the survival of every mammalian species, and logically, they underlie a high level of complexity. Dysfunctions in these processes can lead to infertility. In humans it is a considerable public health problem, affecting up to 15% of couples. Due to the number of factors involved in a successful reproductive process, the mechanistics of infertility are far to being completely understood.

At present, although hundreds of mutant mouse models with reproductive phenotypes have been generated [2] and substantial progress has been made in the identification of genetic causes of human infertility, more than 70% of the cases are still considered as idiopathic [3]. Among these, recurrent spontaneous abortion (RSA) (defined by the occurrence of at least three successive pregnancy losses) affects one to five percent of couples [4]. This pathology can be the result of chromosomal anomalies [5], maternal and fetal structural abnormalities [6], [7], thrombophilic disorders [8] and autoimmune disorders such as the antiphospholipid syndrome [9]. However, in fifty percent of the cases the etiology remains unknown [10], [11]. Up to now, RSA genetic causes have already been explored with variable degrees of success. For instance, in 2006, Kaare et al. analyzed the entire open reading frame of the *Amnionless* gene (*AMN*) in patients affected by RSA but no causal mutations could be identified [12]. More recently, the study of Mercier *et al.* described a statistical association between the p.Val617Phe mutation of the Janus kinase 2 protein and RSA [13]. All in all, the intrinsic difficulty to genetically dissect mammalian reproductive phenotypes, in which hundreds of genes interact into subtle regulatory networks, has not permitted to identify etiological molecular factors that could explain a significant proportion of infertility cases.

In recent years, in order to overcome these constraints we created an original mouse model of interspecific recombinant congenic strains (IRCS) which permit to localize chromosomal regions associated with complex phenotypes (Quantitative Trait Loci or QTL) [14]. This model is composed of 53 strains of mice which harbor, on average, 2% of *Mus spretus* SEG/Pas genome fixed at homozygous state on *Mus Musculus* C57Bl6/J (B6) genomic background. Using IRCS animals we have previously shown that 3 QTL of embryonic lethality mapped on a unique *spretus* fragment in 3 strains, 66H-MMU13, 66H-MMU1 and 135E. The first, *Led1* in 66H-MMU13 strain on the MMU13 (∼2.6 Mb) comprised between the rs120693734 and D13Mit47 polymorphic genetic markers. The second, *Led2* in 66H-MMU1 was analyzed in the present study and the third, *Led3* located on MMU19 in 135E strain encompassing a unique Spretus fragment of 8 Mb located between D19Mit49 and D19Mit137 markers. The 66H-MMU1 strain, which encompasses a unique *spretus* chromosomal fragment located on MMU1 is affected by high levels of embryonic death (24.6%). This strain encompasses a QTL of embryonic lethality (named *Led2*) spreading on 32 Mb and containing 215 genes (143 annotated and 72 predicted) [15].

Here, we present a thorough genetic dissection of *Led2*. For this purpose, we created 15 substrains from 66H-MMU1 animals, which encompass distinct overlapping *spretus* fragments. Using *in vivo* high frequency ultrasonography to follow the embryonic development, we used an approach of type “phenotype/genotype association" to refine this QTL of embryonic death. We identified, into the *Led2* QTL, one region of approximately 6 Mb, *Led2minA*, which has a main effect on the rate of embryonic death. In addition, we pointed out a second region, *Led2minB*, which could also have a small effect on the phenotype, although statistically not demonstrated.

## Materials and Methods

### Ethics Statement

Procedures for handling and experimentation were conducted in accordance with the policies of the Paris Descartes University, the Cochin Institute and the Guidelines for Biomedical Research Involving Animals. The experiments were approved by the departmental veterinary services of Paris (approval number: A75 14-02).

### Animals

The 66H-MMU1 strain was created at the Pasteur Institute (Paris) by successive crosses of the two parental species *Mus musculus* (C57BL6/J) and *Mus spretus* SEG/Pas (originating initially from Spain). The design of these crosses was reported in a previous work [16]. For this study, 15 new recombinant substrains were generated by backcrosses of 66H-MMU1 with C57BL6/J mice. After weaning, 4 weeks aged mice were maintained in an animal facility of the Cochin Institute (Paris) at normal temperature (21–23°C) and 14 h light/10 h dark photoperiods with free access to water and food.

### Microsatellite Genotyping

DNA was extracted from mouse tail fragments by a standard procedure. Eight new microsatellites located on MMU1 (D1Mit439, D1Mit183, D1Mit44, D1Mit383, D1Mit8, D1Mit384, D1Mit255, D1Mit438) were genotyped in order to precise the boundaries of the *spretus* segment present in the 66H-MMU1 genome. Primer microsatellites were retrieved from the Mouse Genome Informatic website (MGI) website of the Jackson Laboratory (www.informatics.jax.org). PCRs were performed using Taq DNA Polymerase (New England Biolabs). PCR products were loaded in a 2% Nusieve, 2% agarose gel (Cambrex Bio Science Rockland, Inc).

### Phenotyping: Ultrasonographic Examinations

The gestation was obtained by crossing each IRCS female with a C57/BL6 male. Each female was used one time to collect phenotypic data from the primo-gestation. For each group one female per gestation and the number of animals studied is always >4. C57/BL6 females from the control group were crossed with C57/BL6 males. Substrain’s phenotyping was carried out at the small animal imaging facility of the Cochin Institute using high frequency ultrasonography (VEVO 770, Visulasonics, Toronto, Canada). Eight to 12 weeks IRCS females were mated with C57BL/6J males, during a period of 2.5 days. Then, female mice were anesthetized with 1.5% of isoflurane in order to achieve ultrasound examination (Minerve Veterinary Equipment, France). Briefly, a chemical hair remover was used to eliminate abdominal hair. Ultrasonographic contact gel was used to ensure contact between the skin surface and the transducer. Body temperature, electrocardiographic and respiratory profiles were monitored using ultrasound device’s integrated heating pad and monitoring device (THM150, Indus Instruments, Webster, TX, USA). Examinations were performed using 2 different high frequency probes depending on the size of the embryos: a 60 MHz transducer for early stages of development (RMV708) and a 40 MHz (RMV704) transducer for late developmental stages.

In order to follow the gestation *in vivo*, three ultrasonographic examinations were performed at three time points (between E7 and E14). During each examination, we assessed the number of implanted embryos in each uterine horn as well as their status (alive or dead) was assessed. For each gestation, the embryonic lethality rate was calculated as the number of resorbed embryos in both horns reported to the total number of implanted embryos.

### RNA Extraction and Gene Expression Arrays

Females from IRCS and B6 strains were crossed with B6 males. Each pregnant female was subjected to ultrasonographic examinations in order to precisely determine the embryonic developmental stage. Female mice were euthanized by cervical dislocation and tissues were taken at E12.5. Total RNA of uterine tissue from six mice of the IRC substrain of interest was extracted using TRIzol Reagent (Invitrogen, Carlsbad, CA, USA) in accordance with the manufacturer's instructions. Similarly, six B6 were used to extract total RNA. In order to duplicate microarray hybridizations of samples from uterine tissues, two pools of three RNA extractions were created for both IRCS and B6 animals. One microgram of RNA from IRC substrains of interest and the B6 controls was used for hybridization on a NimbleGen expression array. cDNA synthesis, DNA end-labeling, hybridization, scanning, and data normalization were performed at the genomic/transcriptomic platform of the Cochin institute. The average fluorescence values for each transcript were collected chromosome per chromosome for each analyzed strain. To evaluate gene expression modifications in IRC strains, these fluorescence levels (considered as expression values) were divided gene per gene by the corresponding ones from B6 which were taken as a reference. The results of the gene expression were deposited at GEO (NCBI) with the accession reference GSE32460.

### cDNA Synthesis by Reverse-transcription and Quantitative RT-PCR

After RNA preparation, the total RNA was treated with DNase I (Invitrogen Life Technologies) for 10 min at room temperature followed by inactivation with EDTA (Sigma-Aldrich). Total uterus RNA was reverse transcribed to obtain cDNA using M-MLV Reverse Transcriptase (Invitrogen, Carlsbad, CA, USA) following manufacturer’s protocols.

Quantitative PCR was carried out using fast SYBR Green Master Mix (Applied Biosystems) and a real time PCR system (Light Cycler 1.5, Roche Diagnostics, Division Applied Sciences, Meylan, France) according to standard PCR conditions. To validate the primers used in qRT-PCR, four pairs of primers were tested for each gene and four housekeeping genes were also tested to choose the reference gene (Table S1 in supplemental data). For quantitative calculations, values were normalized to mouse *Cyclophiline A* expression. Primer sequences are listed in Table S1.

### Statistical Analysis

Results are expressed as mean±SEM calculated from the variation between individual female. The statistical significance of the differences observed between the mean values of IRCS and the control group (C57BL/6J) samples was evaluated by t-test using the Bonferroni-corrected levels. As we used 7 substrains, a p value <0.007 (0.05/7) was considered as significant. Statistically significant results are labeled as follows in all figures: *: p<0.05; **: p<0.01; ***: p<0.001.

## Results

### Creation of 66H-MMU1 Substrains

**Figure 1:**
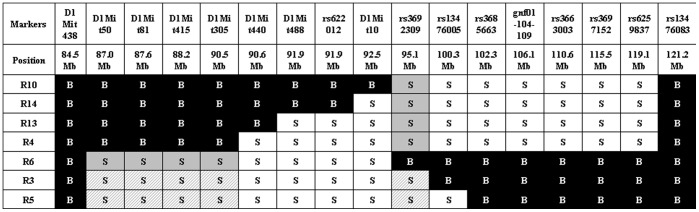
Genomic structure of the IRCS mice in the region of interest of chromosome 1. The map presents the genomic background, spretus or musculus, of the 7 recombinant substrains (Rc) in the chromosomal region corresponding to *Led2* QTL. Recombinant strains were generated at the Pasteur institute (Paris) from 66HMMU1 strain by recombination events inside the MMU1 *spretus* segment. These strains have been genotyped using 24 polymorph markers. Marker positions are given in megabase pairs (Mb). “S" corresponds to the marker in a spretus homozygous form and “B" to the marker in a musculus (B6) homozygous form. The two minimal *spretus* regions (*Led2minA* with main effect and *Led2minB* with probable weak effect) responsible for the phenotype of interest are highlighted in gray and in gray hatched when coexisting in the same substrain.

We started our study using the 66H-MMU1 strain which harbors a high rate of embryonic lethality (24.6%) caused by the *Led2* QTL [15]. This QTL of ∼32 Mb, which was initially delimited by D1Mit50 (87.0 Mb) and rs6259837 (119.1 Mb) markers located on chromosome 1, corresponds to a *spretus* fragment carried by the 66H-MMU1 substrain. However, a uncertainty of ∼6.4 Mb existed at the proximal boundary of this QTL since the interval comprised between D1Mit134 (80.6 Mb) and D1Mit50 (87.0 Mb) markers corresponds to this distance and the breakpoint is located somewhere between these two markers. Indeed, D1Mit134 and D1Mit50 allele markers are of B6 and *spretus* natures respectively (http://www.pasteur.fr/recherche/unites/Gfons/ircs/ircshome.htm). In an attempt to precise the position of the breakpoint, we genotyped 8 novel markers located on this region that permitted to reduce the recombination region to a 2.5 Mb interval comprised between 84.5 Mb and 87 Mb (markers D1Mit438 and D1Mit50 respectively, see Figure 1). Then, we initiated a fine mapping approach using fifteen recombinant substrain issued from 66H-MMU1 animals. In each of these strains, a crossing-over fragmented the original DNA region of *spretus* origin that was initially present in the 66H-MMU1 strain (Figure 1). Among 15 starting strains, seven survived and were available for our study (recombinants, R3, R4, R5, R6, R10, R13 and R14).

### In vivo Phenotyping

**Figure 2:**
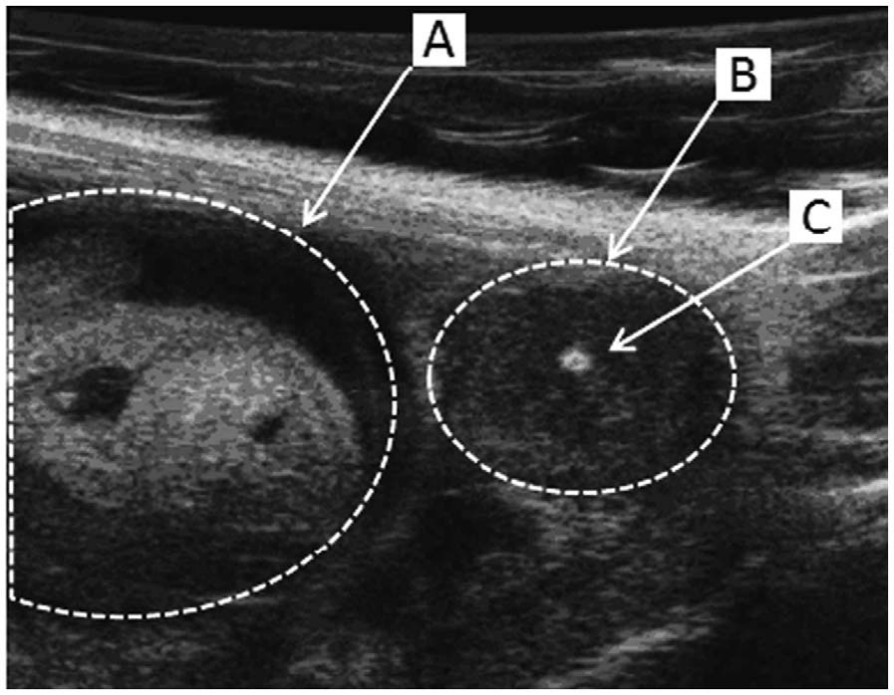
Ultrasound biomicroscopic *in vivo* observation of the embryonic development. During the gestation of B6 and IRC mice the embryonic development was followed up by an *in vivo* ultrasonic method. The viability of developing embryo (A) was assessed by the presence of heartbeats and a positive umbilical cord Doppler. Dead embryos (B) displayed a central highly echogenic zone (C) corresponding to the embryonic resorption.

Females of the different recombinant substrains were crossed with B6 males and their gestation was followed up using *in vivo* ultrasonography as previously described [15]. Animals obtained from these crosses have a placenta which is heterozygous for all the genes located on the fragment of *spretus* origin (B6/SEG), while the uterus was homozygous SEG/SEG for the same *spretus* fragment. The control group was obtained by crossing males and females of the B6 strain. A total of 97 gestations (31 and 66 of B6 and IRCS types respectively) were analyzed. For each gestation, we counted the number of implanted and resorbed embryos during three ultrasonographic examinations. There was no correlation between embryonic death and the position inside the womb, which suggests that the death of one embryo did not have deleterious repercussions on the contiguously implanted structures (Figure 2).

We noted a strong variability in the percentage of embryonic death between the different substrains (Table 1), while apparently the number of implanted embryo was not significantly different. The strains R4, R6, R10, R13 and R14 (group 1) presented a percentage of embryonic death that was not statistically different from that of B6 control animals (10±4%, 19±5%, 9±3%, 12±5% and 16±8% for the five strains, respectively, versus 12±2% in the B6 parent). Note that within this group, R6 is distinguished by the highest rate of embryonic death. By contrast, R3 and R5 strains (group 2) presented a percentage of embryonic death (27±5% and 29±6% respectively) significantly higher than B6 control at p = 0.0013 and p = 0.0045 respectively (Table 1). Since the mean number of implanted embryos was not different between the substrains compared to the B6 control (Table 1), we deduced that the increase in embryonic death observed for R3 and R5 IRCS was caused by post-implantation events.

**Table 1:** Statistical analysis of the embryonic resorption phenotype.

Strains	Number of gestations	Implanted embryos; Mean ± SEM (p value)	Embryonic resorption rate; Mean ± SEM(p value)
B6	31	7.90±0.41	12%±2
R3	13	7.46±0.50 (p = 0.2717; NS)	**27%±5 (p = 0.0013)**
R4	15	7.20±0.71 (p = 0.1845; NS)	10%±4 (p = 0.3021; NS)
R5	4	9.50±0.50 (p = 0.0932; NS)	**29%±6 (p = 0.0045)**
R6	6	8.50±0.99 (p = 0.2848; NS)	19%±5 (p = 0.1158; NS)
R10	8	9.12±0.51 (p = 0.0828; NS)	9%±3 (p = 0.2188; NS)
R13	12	8.83±0.45 (p = 0.1045; NS)	12%±5 (p = 0.4349; NS)
R14	8	8.75±0.70 (p = 0.1759; NS)	16%±8 ((p = 0.2351; NS)

Comparison between IRCs and B6 control using t-test with Bonferroni-corrected level.

(NS: non-significant).

### QTL Fine Mapping

**Figure 3:**
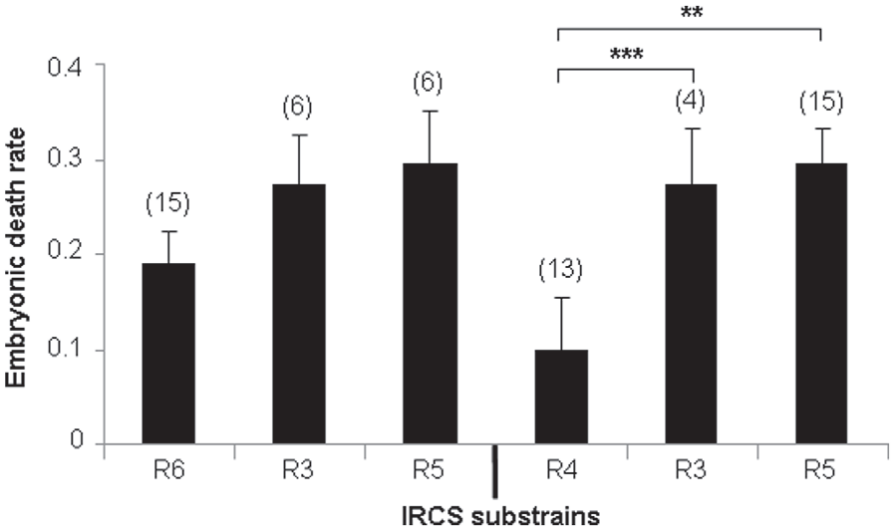
Statistical comparisons between embryonic death rates of IRCS. The mean of embryonic death rate (±SEM) for (n) gestations is presented for four substrains containing different regions of spretus origin: R6 (containing *Led2minA* only), R3 and R5 (containing Led2minA and Led2minB), and R4 (Led2minB only).

In order to refine *Led2* localization, we realized an analysis by genotype/phenotype segregation. R3 and R5 strains (which exhibit the phenotype) shared a large *spretus* region (>84.5 Mb to 90.5 Mb) with the R6 strain (which does not display the embryonic resorption phenotype) and the rest (until <100.3 Mb) is also shared with the other strains (R4, R10, R13 and R14) which are not affected. This configuration suggests that two *spretus* regions, shared by R3 and R5 strains and not present together in the other strains, seem to be necessary to explain the apparition of the phenotype in R3 and R5. We defined a first *spretus* subfragment called *Led2minA* which encompasses D1Mit50 to D1Mit305 region (>84.5 Mb to <90.5 Mb) and a second region called *Led2minB* located at the rs3692309 marker (>92.5 Mb to <100.3 Mb) (see gray boxes in Figure 1). When these two *spretus* regions (*Led2min*) are separated as in R6 (that contains *Led2minA* only) or in R4, R10, R13 or R14 (*Led2minB* only) the phenotype of embryonic death is absent. The presence of the two *spretus* regions seems indispensable to permit the manifestation of the phenotype, it’s the case for R3 and R5 (gray hatched boxes in Figure 1). To statistically prove the presence of these two QTLs, *Led2minA* and *Led2minB* each one responsible for a part of the effect on the phenotype, and an eventual epistatic interaction between these two QTLs able to increase the embryonic death, we compared several recombinants among themselves, R6 bearing *Led2minA*, R4 bearing *Led2minB* and R3 (or R5) bearing these two *spretus* regions. The results of statistical t-tests are shown in Figure 3. When we compared the mean rate of embryonic death between R4 and R3 (or R5), the statistical result (significant difference at P≤0.01) proved the presence of *Led2minA* QTL. By contrast, the comparison between R6 and R3 (or R5), did not statistically indicate the presence of *Led2minB* QTL. However the embryonic death rates of R3 and R5 both have a tendency to be higher than that of R6 (Figure 1) suggesting a possible very small effect of *Led2minB* on the phenotype. In the same way, the difference in embryonic death rate between R4 (*Led2minB* only) and R3 (or R5) was 17%–19% which is comparable to 15%–17% difference between B6 (with no spretus regions) and R3 (or R5) also suggesting a nil or very small effect of *Led2minB*. In consequence this result did not support the presence of an epistatic interaction between *Led2minA* and *Led2minB* regions, but a *Led2minB* additive effect could be revealed by increasing sample size of this “QTL" representative strains. For this raison, the genes present in these two regions, *Led2minA* and *Led2minB* are listed in Table 2.

**Table 2:** Gene list in the minimal *Led2min* region.

Start of the gene	End of the gene	Gene symbol	Gene name
***Led2minA***			
84600220	84718314	*Trip12*	thyroid hormone receptor interactor 12
84718880	84779509	*Fbxo36*	F-box protein 36
84785729	84814108	*Slc16a14*	solute carrier family 16 (monocarboxylic acid transporters), member 14
84906589	84918163	*LOC665317*	
84967431	84988878	*A530032D15Rik*	
84990836	84993050	*A530040E14Rik*	
85035923	85036724	*LOC624074*	
85075992	85092566	*LOC665378*	
85125792	85149592	*LOC620078*	
87313813	87358355	*LOC665338*	
87327496	87345775	*LOC665408*	
87408069	87429975	*Sp110*	nuclear body protein
87431873	87476208	*LOC434484*	
87481184	87532732	*Sp100*	nuclear antigen Sp100 Gene
87548251	87567800	*A630001G21Rik*	
87568115	87625253	*LOC631657*	
87626533	87681536	*Cab39*	calcium binding protein 39 Gene
87725735	87739863	*Itm2c*	integral membrane protein 2C Gene
87759653	87763530	*4933407L21Rik*	
87770707	87792178	*Gpr55*	G protein-coupled receptor 55 Gene
87852883	87855839	*Spata3*	spermatogenesis associated 3 Gene
87877133	87886628	*2810459M11Rik*	
87895789	87970466	*Psmd1*	proteasome (prosome, macropain) 26S subunit, non-ATPase, 1
87930441	87942088	*Htr2b*	5-hydroxytryptamine (serotonin) receptor 2B
87985962	88107425	*Armc9*	armadillo repeat containing 9 Gene
88134638	88138476	*B3gnt7*	UDP-GlcNAc:betaGal beta-1,3-N-acetylglucosaminyltransferase 7
88154437	88156604	*LOC383538*	
88175889	88190626	*Ncl*	nucleolin
88217495	88219312	*Nmur1*	neuromedin U receptor 1
88257499	88259223	*1700019O17Rik*	
88357906	88361869	*Ptma*	prothymosin alpha
88374185	88413672	*Pde6d*	phosphodiesterase 6D, cGMP-specific, rod, delta
88418270	88437671	*Cops7b*	COP9 (constitutive photomorphogenic) homolog, subunit 7b (Arabidopsis thaliana)
88497461	88501743	*Nppc*	natriuretic peptide type C
88535013	88881263	*4930429A22Rik*	Dis3l2 : DIS3 mitotic control homolog (S. cerevisiae)-like 2
88739906	88740882	*LOC620213*	
88917860	88921082	*Akp5*	Alppl2 alkaline phosphatase, placental-like 2
88929174	88932777	*Alpi*	alkaline phosphatase, intestinal
88956178	88959083	*Akp3*	alkaline phosphatase 3, intestine
88978825	88986198	*Ecel1*	endothelin converting enzyme-like 1
89014484	89019578	*1700027L20Rik*	
89021807	89030277	*Chrnd*	cholinergic receptor, nicotinic, delta polypeptide
89036980	89042806	*Chrng*	cholinergic receptor, nicotinic, gamma polypeptide
89045084	89071659	*Eif4e2*	eukaryotic translation initiation factor 4E member 2
89095534	89141962	*Efhd1*	EF hand domain containing 1
89158223	89280317	*Tnrc15*	Gigyf2 GRB10 interacting GYF protein 2
89301371	89306653	*3110079O15Rik*	
89308004	89341517	*Ngef*	
89425720	89428998	*Neu2*	neuraminidase 2
89451549	89551673	*Inpp5d*	inositol polyphosphate-5-phosphatase D
89587241	89623593	*Atg16l1*	autophagy-related 16-like 1 (yeast)
89634850	89676328	*Sag*	retinal S-antigen
89776291	89839722	*Usp40*	ubiquitin specific peptidase 40
89926245	90050168	*Ugt1a7c*	UDP glucuronosyltransferase 1 family, polypeptide A7C
89965979	90050174	*Ugt1a6a*	UDP glucuronosyltransferase 1 family, polypeptide A6A
90031781	90050168	*Ugt1a2*	UDP glucuronosyltransferase 1 family, polypeptide A2
90035905	90036919	*Dnajb3*	DnaJ (Hsp40) homolog, subfamily B, member 3
90094279	90108691	*6430706D22Rik*	
90134452	90220022	*Trpm8*	transient receptor potential cation channel, subfamily M, member 8
90238189	90257609	*Spp2*	secreted phosphoprotein 2
90331041	90341237	*Glrp1*	glutamine repeat protein 1
90529783	90533314	*Arl4c*	ADP-ribosylation factor-like 4C
***Led2minB***			
92505564	92510197	*LOC433332*	
92597263	92674343	*Col6a3*	collagen, type VI, alpha 3
92745512	92780165	*Mlph*	melanophilin
92783513	92784418	*LOC623503*	
92788540	92800026	*Rab17*	RAB17, member RAS oncogene family
92883912	92959324	*Lrrfip1*	leucine rich repeat (in FLII) interacting protein 1
92975508	93001201	*Gm817*	RNA binding motif protein 44
93010446	93054085	*Ramp1*	receptor (calcitonin) activity modifying protein 1
93080775	93082727	*LOC623550*	
93080971	93116330	*Ube2f*	ubiquitin-conjugating enzyme E2F (putative)
93128743	93151480	*Scly*	selenocysteine lyase
93152480	93178709	*Gm556*	espin-like
93181478	93192810	*4631423F02Rik*	(Klhl30) kelch-like 30 (Drosophila)
93196865	93204619	*BC056923*	(Fam132b) family with sequence similarity 132, member B
93206236	93229189	*Ilkap*	integrin-linked kinase-associated serine/threonine phosphatase 2C
93235288	93238577	*1700020N18Rik*	
93241888	93243628	*Hes6*	hairy and enhancer of split 6 (Drosophila)
93246387	93289702	*Per2*	period homolog 2 (Drosophila)
93325082	93358670	*Traf3ip1*	TRAF3 interacting protein 1
93370970	93390034	*Asb1*	ankyrin repeat and SOCS box-containing 1
93631882	93678433	*Twist2*	twist homolog 2 (Drosophila)
93763139	93978799	*Hdac4*	histone deacetylase 4
94270113	94304164	*Ndufa10*	NADH dehydrogenase (ubiquinone) 1 alpha subcomplex 10
94310086	94311025	*Olfr1416*	olfactory receptor 1416
94321223	94322159	*Olfr1415*	olfactory receptor 1415
94341493	94342432	*Olfr1414*	olfactory receptor 1414
94403578	94404550	*Olfr1413*	olfactory receptor 1413
94418737	94419703	*Olfr1412*	olfactory receptor 1412
94426926	94427898	*Olfr1411*	olfactory receptor 1411
94438244	94439213	*Olfr1410*	olfactory receptor 1410
94450313	94451315	*Olfr12*	olfactory receptor 12
94467550	94472391	*Myeov2*	myeloma overexpressed 2
94474623	94479247	*Otos*	otospiralin
94662091	94690602	*Gpc1*	glypican 1
94700534	94733312	*Ankmy1*	ankyrin repeat and MYND domain containing 1
94737394	94739026	*0710001B24Rik*	(Dusp28) dual specificity phosphatase 28
94741810	94750986	*Rnpepl1*	arginyl aminopeptidase (aminopeptidase B)-like 1
94764813	94778354	*Capn10*	calpain 10
94809526	94815938	*Gpr35*	G protein-coupled receptor 35
94836739	94842672	*Aqp12*	aquaporin 12
94848683	94932228	*Kif1a*	kinesin family member 1A
94965650	94988099	*Agxt*	alanine-glyoxylate aminotransferase
94981761	94991354	*2310007B03Rik*	
94999130	95061480	*E030010N08Rik*	
95066246	95131471	*Sned1*	sushi, nidogen and EGF-like domains 1
95129616	95136276	*Mterfd2*	MTERF domain containing 2
95139842	95167980	*Pask*	PAS domain containing serine/threonine kinase
95174050	95198024	*Ppp1r7*	protein phosphatase 1, regulatory (inhibitor) subunit 7
95204301	95233908	*Tmem16g*	Ano7: anoctamin 7
95236345	95309214	*Hdlbp*	high density lipoprotein (HDL) binding protein
95252098	95252952	*LOC621682*	
95309449	95340136	*sept-02*	septin 2
95342530	95452188	*Farp2*	FERM, RhoGEF and pleckstrin domain protein 2
95452309	95466056	*Stk25*	serine/threonine kinase 25 (yeast)
95516099	95526168	*Bok*	BCL2-related ovarian killer protein
95535796	95585244	*Thap4*	THAP domain containing 4
95585438	95619935	*Atg4b*	autophagy-related 4B (yeast)
95630900	95632281	*Dtymk*	deoxythymidylate kinase
95634370	95652507	*Ing5*	inhibitor of growth family, member 5
95655698	95682580	*D2hgdh*	D-2-hydroxyglutarate dehydrogenase
95691749	95706900	*Gal3st2*	galactose-3-O-sulfotransferase 2
95792483	95816522	*LOC666009*	
95820914	95841947	*LOC619597*	
95850898	95858740	*Neu4*	sialidase 4
95868710	95882959	*Pdcd1*	programmed cell death 1
97144149	97165471	*2310044D20Rik*	Fam174a : family with sequence similarity 174, member A
97418087	97498000	*St8sia4*	ST8 alpha-N-acetyl-neuraminide alpha-2,8-sialyltransferase 4
98475880	98476840	*Gm1833*	predicted gene 1833
98648136	98702541	*Slco4c1*	solute carrier organic anion transporter family, member 4C1
98702644	98708307	*9530060I07*	
98736581	98827966	*Slco6b1*	solute carrier organic anion transporter family, member 6b1
98889854	98958709	*Slco6c1*	solute carrier organic anion transporter family, member 6b1
98993265	99132680	*LOC634331*	
99176834	99309408	*LOC634346*	
99474308	99492382	*D1Ertd622e*	DNA segment, Chr 1, ERATO Doi 622, expressed
99536564	99591955	*Hisppd1*	Ppip5k2: diphosphoinositol pentakisphosphate kinase 2
99600605	99623375	*4930429M06Rik*	Gin1: gypsy retrotransposon integrase 1
99651499	99925919	*Pam*	peptidylglycine alpha-amidating monooxygenase
100251529	100339787	*Slco6d1*	solute carrier organic anion transporter family, member 6d1

### Is Placenta or Uterus Responsible for the Led2min Effect?

**Figure 4:**
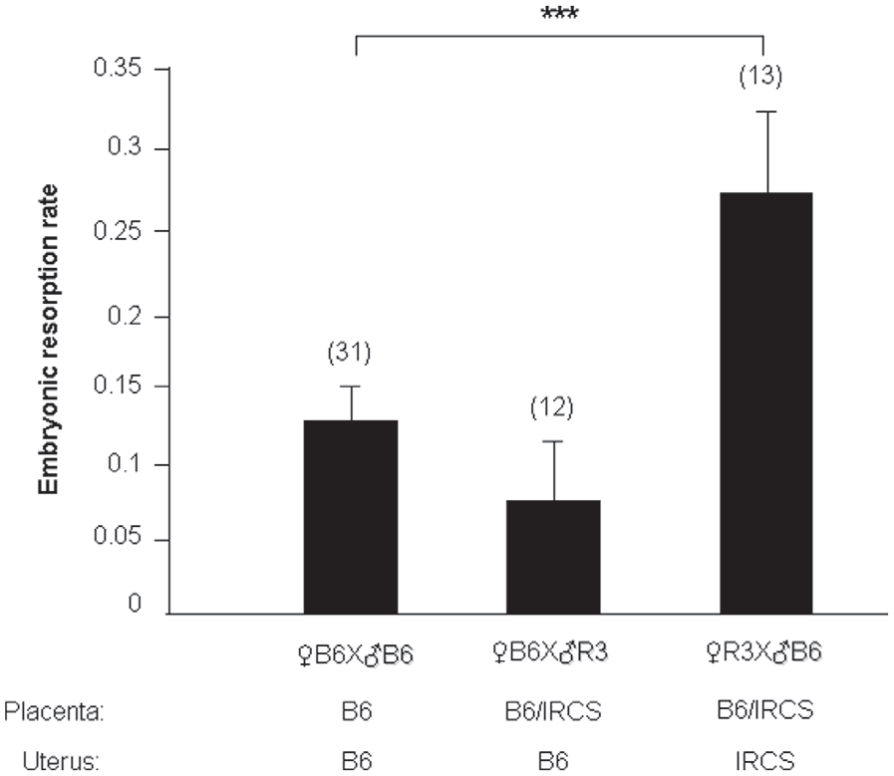
Embryonic resorption rate in function of the type of crossing realized with IRCS and B6 mice. The results of different crosses (♀IRCS × ♂B6, ♀B6 × ♂IRCS and ♀B6 × ♂B6) are presented as the average (±SEM) of the embryonic resorption rate for (n) gestations.

Since R3 and R5 mice did not harbor any obvious developmental anomaly or pathology, excepting for some embryonic death, it was reasonable to suspect that placental and/or uterine dysfunctions could be responsible for the embryonic lethality increase. Thus, we initiated a genetic approach in order to identify in which of these two organs dysfunction could be related with the phenotype. In the previous set of experimental crosses, IRCS females were mated with B6 males. Genetically, this permitted the co-existence of heterozygous foeto-placental alleles (B6/SEG) and homozygous uterine alleles (SEG/SEG) within the same genomic region (*spretus* fragment). Conversely, we performed reverse crosses (♀B6 × ♂IRCS), giving a heterozygous placenta for the genes of the fragment, but a B6 homozygous uterus. In this situation, the would-be disorders ought to find their origin exclusively from a placental-fetal/embryonic defect, caused by the *spretus* state of the MMU1 fragment, but not from a B6 womb defect. In this optic we realized the cross ♀B6 with ♂R3 (IRCS group2). We observed that the mean of embryonic resorption rate (±SEM) was 0.07±0.04 and not significantly different from the control (♀B6 × ♂B6: 0.12±0.02; p = 0.118) whereas the inverse crossing, leading also to a heterozygous foeto-placental complex implanted in homozygous spretus uterus (R3), produced a significantly higher embryonic resorption rate (0.27±0.05, p = 0.001) (Figure 4). From this last observation, we concluded that a uterine dysfunction is very likely at the basis of the observed phenotype.

### Assessing of Differentially Expressed Genes in Uterus

We analyzed the expression level of uterine genes in pregnant R3 females compared to those from B6 control animals at E12.5, an important time point when most resorption occurred during our study. For this purpose, we hybridized cDNA synthesized from RNA uterine tissue to Nimblegen mouse microarrays. The Nimblegen arrays interrogated a total of 25,631 mouse transcripts. Gene expression levels were quantified by fluorescence intensity assessing and ranged from 20 to >50,000 arbitrary units of fluorescence (AUF) (mean value ∼5,250 AUF). These results were highly reproducible since they showed strong correlations between experimental duplicates (r = 0.967 for B6 and 0.983 for R3). Thus, for subsequent analysis, we took the average of both values for each transcript. We first focused on transcripts with fluorescence levels higher than 100 AUF, we assumed that values under this threshold were very close to background signals and, with this threshold, we selected 18,085 transcripts (70.6% of the total). We considered a gene as differentially expressed if a two-fold difference of expression (up or down) was observed. Consequently, 3,436 (19% of the expressed uterine genes) transcripts were modified in R3 uterus when compared to those expressed in B6 (Table 3). A similar number of repressed and induced genes was observed (10.9% and 8.1%, respectively). This deregulation was identified over all the genome. However a significantly higher proportion of genes were deregulated when only the MMU1 *spretus* fragment or the two *Led2min* regions were considered (Table 3).

**Table 3:** Number of transcripts modified in uterus of R3 IRCS compared to C57BL6/J.

Genomic region	Expressed Transcripts (>100 AUF)	Deregulated transcripts (2-fold threshold)
Total genome	18,085	3,436 (19%)
*Spretus* fragment in MMU1	148	53 (36%)
*Led2minA* (84.5–90.5 Mb)	55	24 (44%)
*Led2minB* (95.1 Mb to <100.3 Mb)	25	8 (32%)

In order to validate the differential gene expression obtained by the microarray analysis, we checked 7 genes of the *Led2minA* QTL region by quantitative RT-PCR. As shown in Table 4 we obtained a very good agreement between microarray and qPCR results.

**Table 4:** Microarray validation by RT-QPCR on 7 genes of the QTL region.

Gene symbol	Microarrays		RT-PCR		
	Expression level in B6 uterus (AUF)	Ratio of expressionR3/B6	Ratio of expressionR3/B6	% PCR efficiency	R-square values
*Ncl*	1555	0.16	0.42249197	101%	0.9986
*Cab39*	1800	4.5	5.43093265	102%	0.99
*Psmd1*	34109	0.90	0.69502902	104%	0.9934
*Cops7b*	12526	0.62	0.66533846	105%	0.9999
*Eif4e2*	652	0.5	0.50713771	105%	0.9913
*Usp40*	2154	0.21	0.39715333	100%	0.9992
*Trip12*	12096	0.77	0.60419569	101%	0.9991
*Cyclophilin A* (Reference gene)	38918	0.95		95%	0.9994

Considering that the uterine dysfunction can take its origin from a deregulation of the gene expression or/and non-synonymous coding polymorphisms accumulated during independent evolutive processes of *Mus musculus* and *Mus spretus* species, we listed the genes of the *Led2min* QTL corresponding to these criteria and thus potentially involved in embryonic resorption. Finally we considered the transcripts with an expression level >500 AUF and either exhibiting a deregulation (R3/B6 expressional ratio >2 or <0.5) and the presence of non synonymous polymorphisms between *Mus musculus* and *Mus spretus* (provided by SANGER database: http://www.sanger.ac.uk/) (Table 5).

**Table 5:** Genes of *Led2min* region expressed in uterus (>500 AUF) and displaying a deregulation (R3/B6 ratio) and/or non synonymous SNP.

Gene symbol	Expression level in B6 uterus (AUF)	SNP number	Ratio of expression R3/B6
***Led2minA***			
*Trip12*	12096	4	0.77
*Fbxo36*	992	6	0.12
*A530040E14Rik*	521	0	0.28
*Sp110*	18202	ND	0.52
*SP100*	3798	19	0.55
*Cab39*	1800	0	4.5
*Itm2c*	13239	1	0.44
*Spata3*	996	7	1.33
*2810459M11Rik*	1922	4	0.24
*Psmd1*	34109	2	0.90
*Htr2b*	1982	4	0.13
*Ncl*	1555	5	0.16
*Pde6d*	9972	0	0.15
*Cops7b*	12526	1	0.62
*Eif4e2*	652	1	0.50
*Ngef*	1627	4	0.54
*Usp40*	2154	7	0.21
*Glrp1*	517	9	0.11
*Arl4c*	10043	0	0.37
***Led2minB***			
*Col6a3*	20317	28	2,01
*Mlph*	3388	10	0,48
*Rab17*	2539	4	0,34
*Lrrfip1*	14838	14	1,2
*Ramp1*	13416	7+1 stop lost	0,78
*Ilkap*	20118	2+1 slpice site	0,78
*Hes6*	12151	1	0,57
*Per2*	3786	10	0,89
*Traf3ip1*	814	9	0,66
*Asb1*	4667	1	0,57
*Ndufa10*	32815	2	0,87
*Myeov2*	14896	5	0,73
*Gpc1*	1912	1	0,8
*Rnpepl1*	960	1	0,69
*Sned1*	4230	9	3,51
*Mterfd2*	24052	7	1,32
*Ppp1r7*	8390	8	0,63
*Hdlbp*	25050	2	1,06
*Farp2*	634	20	0,74
*Thap4*	2169	1	0,77
*Atg4b*	678	3	0,72
*Dtymk*	5044	1	0,41
*D2hgdh*	945	3+1 splice site	0,44

ND: Not determined.

Then, we searched whether deregulated transcripts could be grouped into functional clusters using DAVID database [17], considering 1758 transcripts with a threshold of >500 AUF in the expression level. This analysis led to the identification of five functional groups of genes and signaling pathways that were deregulated, such as ribosome protein genes (p value: 0.00054), endocytosis process (p value: 0.0027), VEGF (vascular endothelial growth factor) signaling (p value: 0.0078), chemokine interactions (p value: 0.011) and mTOR (mammalian Target Of Rapamycin) signaling pathway (p value: 0.014).

## Discussion

In the reproductive processes, as in others, hundreds of genes interact into subtle regulatory networks, and this complexity does not permit to easily identify the molecular factors of dysfunctions leading to infertility cases. Moreover, when our interest is turned towards the human clinic, the study of factors involved in reproductive defects is particularly challenging due to obvious ethical constraints, which rends obligatory the use of animal models. However although hundreds of mutant mouse models with infertility/hypofertility phenotypes have been generated [2], the genetic causes of infertilities are far from being elucidated in their whole [3]. This is the case for the RSA pathology which affects a non negligible percentage of the population (1 to 5%) and for which the genetic origin(s) is still little documented. In the aim to identify new genes responsible for embryonic lethality, we used a mouse model of interspecific recombinant congenic strains (IRCS). Although during the gestation/pregnancy development, mice and humans do not establish exactly the same system of placentation, similarities strong enough between these two species exist, making the mouse model useful to identify genes involved in humans. Indeed, in several mouse models involving the complement system [18], [19], it has been clearly shown that there is a continuum between embryo resorption and placental diseases, since the same mice have these resorption and preeclampsia-like symptoms [20]. It is also known that C3 defects are clearly linked with VEGF defects, thus inducing defective placentation, leading in the most extreme cases to embryonic death. Therefore, mouse models of embryo resorption via known deregulations of complement system are proved to be suitable models of the human continuum placental vascular disease-spontaneous resorption. A well studied mouse model of immunologically mediated peri-implantation pregnancy loss that shares features with human recurrent miscarriage is derivated from DBA/2-mated CBA/J mice (CBA/J × DBA/2) [21], [22], [23]. Indeed embryos derived from mating CBA/J females with DBA/2 males showed an increased frequency of resorption (29.4±6.5%), more than three times greater than that seen within these and other strains or strain combinations (CBA/J × CBA/J: 8.9±5.1%; CBA/J × BALB/c: 8.2±5.6%; DBA/2 × DBA/2: 8.5±6.6%; n = 6–32 mice/group; CBA/J × DBA/2 vs. others, p<0.01) [18]. Spontaneous resorption in the CBA/J × DBA/2 model is attributed to NK cells, macrophages, and Th1-type cytokines. and represent a rejection of the semiallogeneic fetoplacental [24]. Murine resorptions are characterized by focal necrosis at the junction of the fetal trophoblast with decidua, an infiltrate of polymorphonuclear leukocytes (with some lymphocytic cells) at sites of necrosis and along the walls of large vessels in decidua, and by thrombosis and hemorrhage [25], [26], [27], [28]. Infiltration begins on day 6.5 of gestation, 2 days after implantation has occurred, and abortions begin after day 8.5 of pregnancy [24], [28].

In the present work we used a mouse model including interspecific recombinant congenic strains (IRCS). The originality of this whole model is based on the presence of a small homozygous fragments of *Mus spretus* genome fixed on a *Mus musculus* B6 genetic background [16]. Thus, a strain differs of each other and from the B6 parental strain by the *spretus* segments. *Mus musculus* and *Mus spretus* diverged ∼2 million years ago meaning that the association of their two genomes has the potential to lead to genetic incompatibilities [14]. Using this model, in past studies, we were able to localize various QTL modulating male and female reproductive processes. We identified a QTL responsible for ∼25% of the embryonic resorptions present in the IRCS-66H-MMU1 strain containing a solely chromosomal fragment of *spretus* origin located on MMU1. This QTL was called *Led2* and has been mapped to an interval of 32 Mb which contains 215 genes [15]. The aim of the present study was to redefine this region and to identify candidate genes potentially involved in embryonic lethality.

To accomplish the fine mapping of this QTL, we generated recombinant substrains from 66H-MMU1 by backcrosses, each of them presenting a unique sub-fragment of the *Led2* QTL. Each recombinant substrain females were crossed with B6 males, resulting in a fetus/placenta complex with heterozygous B6/SEG genes (at the *Led2* locus) and uterine homozygous *spretus* genes (at the *Led2* locus). During each gestation, the substrains were phenotyped *in vivo* by ultrasonography. This non-invasive technology, based upon a high frequency ultrasound device [29] allows *in vivo* real time high resolution observations of embryonic development [15], [30] and resorption (∼70 µm and ∼40 µm lateral and axial resolution, respectively) and permits to carry out longitudinal analysis of gestation. We observed an increase of the embryonic death rate in R3 and R5 substrains (Group 2). The analysis of the genotype/phenotype segregation allowed us to determine two reduced QTL regions (*Led2minA* and *Led2minB*) of approximately 6 Mb each, present together in spretus version only in R3 and R5 strains. In the other recombinant substrains which have not the phenotype, the one or the other of the region is present but not the two regions together. So we defined the first reduced spretus region called *Led2minA* which encompasses D1Mit50 to D1Mit305 region (>84.5 Mb to 90.5 Mb) and the second called *Led2minB* located at the rs3692309 marker (>92.5 Mb to <100.3 Mb). Our statistical analysis succeeded in proving the presence of *Led2minA* QTL responsible for a main effect on embryonic death but it failed it for *Led2minB*. However, notable differences between the embryonic death rates of certain strains (R6 compared to R3 or R5) led to suppose that this latter region could also have a small effect in the phenotype. Taken together, these data did not support the presence of an epistatic interaction between *Led2minA* and *Led2minB*.

Reverse crosses using IRCS Group 2 males and B6 females revealed that the genes expressed at heterozygous state in the placental tissues are not deleterious for the gestation. Therefore, we deduced that the high rate of embryonic death occurring during the gestation resulted from dysfunction of genes expressed in the uterine tissue. This is in accordance with the normal embryonic development observed in group 2 IRCS females. Then, we carried out a microarray analysis searching to identify uterine deregulated genes in IRCS animals from Group 2. Although we observed deregulated genes located in all chromosomes (19%) we noticed that those situated on the *spretus* fragment were preferentially modified (∼40%). This concentration of deregulated genes located on the *spretus* fragment has already been reported in a previous study of our group performed on testis transcriptome [31]. It has been showed that at genomic scale differential SNPs between *Mus musculus* and *Mus spretus* are frequent since they appear, in average, every 100 bp. When located on the promoter regions of *spretus* origin, these nucleotide substitutions could modify the transactivation/transrepression properties of transcription factors of C57BL/6J nature, thus modifying the *spretus* gene expressions. Additionally, dysfunctions leading to embryonic death could result from non-synonymous coding polymorphisms, accumulated during evolution in the *spretus* genome. These phenomena should be originated from evolution of separated genomic regions that produces transcription factors/DNA (“transcriptomic shock") and/or protein-protein (“proteomic shock") incompatibilities [32].

Focusing on genes of the *Led2minA* QTL and applying filters from bioinformatics databases, bibliography and our own results, we propose a selection of 7 genes (*Trip12*, *Cab39*, *Psmd1*, *Ncl*, *Cops7b*, *Eif4e2* and *Usp40)* as putative actors of the embryonic death. These genes play a role in VEGF signaling, mTOR signaling and ubiquitine/proteasome-protein degradation pathway. Their effects could be reinforced by a small participation of genes situated *on Led2minB* region and which could act in the same signaling pathways (*Asb1, Traf3ip1*, *Ramp1* and *Col6a3*).

*Trip12*, *Psmd1*, *Cops7b* and *Usp40* from *Led2minA* and *Asb1* from *Led2minB* are involved in protein degradation process through the ubiquitin-proteasome pathway. *Trip12* exerts a ligase activity related to ubiquitination [33], *Usp40* functions as a deubiquitination enzyme in the same degradation pathway [34] and *Asb1* is a member of the ankyrin repeat and SOCS box (ASB) family. These family proteins interact with Cul5-Rbx2 to form E3 ubiquitin ligase [35]. *Psmd1* is a component of the 26S proteasome. Cops7b is a subunit of the eight-subunit heteromeric Cop9 signalosome complex. Genetic invalidations of some *Cop9* subunits have been associated with developmental defects of post-implantation embryos [36], [37], [38]. Additionally, *Usp40* functions as a deubiquitination enzyme in the same degradation pathway [34]. *Asb1* is a member of the ankyrin repeat and SOCS box (ASB) family. These family proteins interact with Cul5-Rbx2 to form E3 ubiquitin ligase [35].

In the same manner, *Led2minA Ncl* gene and *Led2minB Ramp1* and *Col6a3* genes are involved in angiogenesis. *Ncl* encodes nucleolin and treatment of endothelial cells with anti-nucleolin antibody induces apoptosis of these cells [39]. Moreover, nucleolin associates with VEGF-C62 and can be potentially involved in epithelial cell adhesion and proliferation [39], [40]. Concerning *Ramp1* gene, RAMP1 (receptor activity modifying protein) forms a functional receptor for CALCA (Calcitonin gene-related peptide) which is a proangiogenic growth factor in the human placental development and plays a critical role in embryonic development and fetal growth [41]. Concerning the *Collagen typeVI a3* gene, COL IV is a main endometrial extracellular matrix component, and an abnormal increased deposition of collagen might impair uterine function, possibly by interfering with vascularization or retarding remodeling events at implantation [42].

Finally, *Cab39* (also called *Mo25*) and *Eif4e2* from *Led2minA* and *Traf3ip1* from *Led2minB*, participate in the mTOR signaling pathway, a regulatory step of protein synthesis and growth. *Cab39* effect has been described upstream of mTOR activation while *Eif4e2* is a downstream signaling target involved in translation initiation. Interestingly, *Mtor* genetic disruption in mice leads to early embryonic death [43]. Homozygous *Traf3ip1* (Tumor necrosis factor alpha receptor 3 interacting protein 1) mutant mice are not viable. *Traf3ip1* mutant mouse line was generated and the enlarged mutant cell size in culture was associated with elevated basal mTOR pathway activity [44]. Otherwise, mTOR pathway is implicated on the VEGF pathway activation. It is worth noting that the VEGF and mTOR pathways have been identified as significantly deregulated in our functional clustering analysis.

### Conclusions

We used an *in vivo* approach of the embryonic development on a mouse IRCS model to refine a chromosome 1 region (*Led2*) responsible for embryonic death. The present study succeeded in fine-mapping *Led2minA* QTL which has a main effect on the embryonic death (about 30%) and pointed out a second region *Led2minB* which could have a minor effect on the same phenotype. Collecting and analyzing experimental, bioinformatics and literature data on the expression and function of genes present in the two regions (*Led2minA and Led2minB*), we propose 7 genes from *Led2minA* that could be related with the phenotype. It appears that the vascularization could be the common denominator at these categories of genes involving angiogenesis and the fluidity of the extracellular matrix. The actual identification of the gene(s) involved in this phenotype will necessarily pass through further molecular approaches. An important outcome of this study is the possibility to evaluate novel promising candidates of RSA in humans [13]. This might contribute to elucidate the molecular basis of this multifactorial and complex human disorder and to propose new diagnostic markers.

## Supporting Information

Table S1 **Sequences of used real time RT-PCR primers.**
(DOC)
