# Supplementary material for: Refined Mapping of a Quantitative Trait Locus on Chromosome 1 Responsible for Mouse Embryonic Death
Source: PLoS One. 2012 Aug 16;7(8):e43356. doi: 10.1371/journal.pone.0043356 (PMC3420870; doi:10.1371/journal.pone.0043356)
Supplement: Table S1 — Sequences of used real time RT-PCR primers. (DOC) [file pone.0043356.s001.doc]

**Table S1: Sequences of used real time RT-PCR primers.**

| *Cyclophilin A* | Forward | 5’-GTCAACCCCACCGTGTTCTT-3’ |
| --- | --- | --- |
|  | Reverse | 5’-CTGCTGTCTTTGGGACCTTGT-3’ |
| *Usp40* | Forward | 5’-GGAACAGCAGTCCAGCACTC-3’ |
|  | Reverse | 5’- GTGAGCAGTTGGTGGAGTCA-3’ |
| *Psmd1* | Forward | 5’-GATCCAGGCACAGAAGCAAT-3’ |
|  | Reverse | 5’-AGGAGGACAACCTGCTGATG-3’ |
| *Ncl1* | Forward | 5’-TAGGTTTGCCATGTGGGTTC-3’ |
|  | Reverse | 5’-TTAAGTAACCTTTCCTACAGTGCAAC-3’ |
| *Trip12* | Forward | 5’-GAGGTGGCTGATTTTGAAGC-3’ |
|  | Reverse | 5’-TGTGGCTGCTGGTAGTTCAC-3’ |
| *Eif4e2* | Forward | 5’-AAAACTTCCAGAACTGCTCCA-3’ |
|  | Reverse | 5’-AGAGCAGTGGCAAGAGGAAG-3’ |
| *Cops7b* | Forward | 5’-ACGGCCAATGCAGAAATC-3’ |
|  | Reverse | 5’-CTGTGCTGCTGAAAGACCTG-3’ |
| *Cab39* | Forward | 5’-TCTGTCCAACAGCAGCTCAC-3’ |
|  | Reverse | 5’-GCTCAGTGCAGAATTTTTGG-3’ |
